# Increasing the diagnostic yield of exome sequencing by copy number variant analysis

**DOI:** 10.1371/journal.pone.0209185

**Published:** 2018-12-17

**Authors:** Daniel S. Marchuk, Kristy Crooks, Natasha Strande, Kathleen Kaiser-Rogers, Laura V. Milko, Alicia Brandt, Alexandra Arreola, Christian R. Tilley, Chris Bizon, Neeta L. Vora, Kirk C. Wilhelmsen, James P. Evans, Jonathan S. Berg

**Affiliations:** 1 Department of Genetics, University of North Carolina, Chapel Hill, NC, United States of America; 2 Department of Pathology and Laboratory Medicine, University of North Carolina, Chapel Hill, NC, United States of America; 3 Department of Pediatrics, School of Medicine, University of North Carolina, Chapel Hill, NC, United Sates of America; 4 Renaissance Computing Institute, University of North Carolina, Chapel Hill, NC, United States of America; 5 Department of Obstetrics and Gynecology, University of North Carolina, Chapel Hill, NC, United States of America; German Cancer Research Center (DKFZ), GERMANY

## Abstract

As whole exome sequencing (WES) becomes more widely used in the clinical realm, a wealth of unanalyzed information will be routinely generated. Using WES read depth data to predict copy number variation (CNV) could extend the diagnostic utility of this previously underutilized data by providing clinically important information such as previously unsuspected deletions or duplications. We evaluated ExomeDepth, a free R package, in addition to an aneuploidy prediction method, to detect CNVs in WES data. First, in a blinded pilot study, five out of five genomic alterations were correctly identified from clinical samples with previously defined chromosomal gains or losses, including submicroscopic deletions, duplications, and chromosomal trisomy. We then examined CNV calls among 53 patients participating in the NCGENES research study and undergoing WES, who had existing clinical chromosomal microarray (CMA) data that could be used for validation. For unique CNVs that overlap well with WES coverage regions, sensitivity was 89% for deletions and 65% for duplications. While specificity of the algorithm calls remains a concern, this is less of an issue at high threshold filtering levels. When applied to all 672 patients from the exome sequencing study, ExomeDepth identified eleven diagnostically relevant CNVs ranging in size from a two exon deletion to whole chromosome duplications, as well as numerous other CNVs with varying clinical significance. This opportunistic analysis of WES data yields an additional 1.6% of patients in this study with pathogenic or likely pathogenic CNVs that are clinically relevant to their phenotype as well as clinically relevant secondary findings. Finally, we demonstrate the potential value of copy number analysis in cases where a single heterozygous likely or known pathogenic single nucleotide alteration is identified in a gene associated with an autosomal recessive condition.

## Introduction

The relatively low cost of whole exome sequencing (WES) and the theoretical ability to detect deleterious genetic anomalies in nearly the entire coding region of the genome make WES an appealing approach to the clinical diagnosis of patients with a broad spectrum of phenotypes [1]. However, even after a thorough analysis of rare coding SNVs and indels in known disease genes, most patients with suspected genetic conditions are left without an explanation for their symptoms [2,3]. These cases may be negative for a number of reasons including non-genetic etiologies, lack of knowledge about the genes that cause different disease phenotypes, or in some cases a deletion or duplication of genomic information not routinely detectable by WES variant calling. While most of these alternative explanations are impossible to adjudicate without additional testing, CNV (copy number variation) detection is possible using only WES data. However, such analysis presents considerable challenges.

Many researchers have created methods to detect large gains or losses of genetic information from WES data [4–8]. Utilizing WES read depth data, read count distribution models can be generated to infer the copy number states of exonic regions of the genome. This approach relies on the assumption that the number of reads covering a region will be directly proportional to the number of copies of that locus present in the sample. While many such methods exist, their clinical implementation with WES is not yet routine.

Validated approaches to CNV calling could have a significant impact on the diagnostic rate of WES testing and make WES analysis a logical first-line, stand-alone diagnostic test for many conditions. In children with intellectual disability or developmental delay, an estimated 14% of all cases have pathogenic CNVs larger than 400kb [9]. In theory, CNV detection by WES methods could be more sensitive to smaller CNV than chromosome microarray testing, the current standard for detecting submicroscopic CNVs is as small as ~40 kb [10] (although newer exon arrays may improve this resolution). In contrast, a reasonable lower limit for CNV detection from WES methods could be ~200bp or around the size of one exon [6,11] making CNV detection by WES a promising alternative. On the other end of the spectrum, WES coverage data can also be used to identify whole chromosome aneuploidy. Given the potential clinical significance of whole chromosome abnormalities, including mosaic aneuploidies, which may be less clinically recognizable than full aneuploidies yet clinically significant [12], adding this capability to a WES read depth-based method further increases the capability of WES testing to identify genomic variants across the entire size spectrum. Finally while whole genome sequencing has tremendous potential for the identification of CNVs and may one day supplant both WES and microarray analysis, it will likely remain substantially more expensive than WES for the foreseeable future.

In this manuscript, we describe the analysis of WES data to identify clinically relevant CNV and aneuploidy, and we compare the performance of the CNV calling algorithm, ExomeDepth, against clinical microarray data. Our results indicate that there are still limitations of CNV calling from WES data, such that chromosomal microarray will likely remain the gold standard for clinical CNV testing for now. However if incorporated into WES analysis routinely, when high-confidence clinically relevant CNVs are detected in WES data, such analysis may increase diagnostic yield and obviate the need for further testing, such as microarray analysis.

## Methods

### Participants

Exome sequencing was conducted as part of the North Carolina Clinical Genomic Evaluation of Next-Generation Exome Sequencing (NCGENES) study [13]. NCGENES assessed the clinical implementation of WES in people with a broad range of phenotypes including cancer, intellectual disability, cardiomyopathy, retinal dystrophies and many other phenotypes. Patients with a definitive explanation for their phenotype, including pathogenic CNVs detected by CMA testing, were not eligible for NCGENES enrollment. The NCGENES study was approved by the Institutional Review Board of the University of North Carolina at Chapel Hill. Formal written consent was obtained for all participants during an in-person visit with a genetic counselor. Parents or guardians provided written consent for child participants or adults with intellectual disability. Assent was obtained from minors when appropriate.

### Sequencing and informatics

WES sequencing capture and library preparation was carried out according to manufacturer’s guidelines using the Agilent SureSelect XT Target Enrichment System (Santa Clara, CA) with Human All Exon V4 and V5 from peripheral blood specimens. DNA fragmentation was performed using a Covaris E220 sonicator, producing DNA fragment sizes of approximately 200–250 base pairs that were optimal for downstream steps in the library preparation and exome capture workflow. Sequencing was performed on a HiSeq2000 or HiSeq2500 at the UNC High Throughput Sequencing Facility. Mean read depth was 62x. Mapping and variant calling were carried out according to the Broad Institute’s best practices using BWA and GATK as previously described [14].

ExomeDepth, a freely available R package, was used to detect copy number variants from WES read depth data [11]. Expected read counts were modeled for each sample over 100 bp windows using sequencing coverage information from the NCGENES participants. In-house python scripts were used to annotate CNV calls by incorporating CNV data from ISCA (International Standards for Cytogenic Arrays) [15] and DGV (Database of Genomic Variants) [16] and gene annotation information using Refseq and OMIM [17]. Bayes Factors, a likelihood ratio of CNV probability to normal copy number probability, were calculated by ExomeDepth and used to aid in CNV call adjudication. ExomeDepth used in conjunction with the Bayes factor score has been shown to have a higher true positive to false positive ratio than other CNV detection algorithms [8].

The copy number of each chromosome was predicted by taking a ratio of an adjusted chromosomal read count per sample to the mean number of chromosomal reads across all samples. Adjusted read counts were calculated by counting reads only in WES capture regions and then normalized so that the total number of reads per sample was the same across all samples. Because the collection of sample ratios for each respective chromosome followed approximately normal distributions, outliers were detected using the Grubbs outlier test.

### Analysis of WES data

A pilot cohort consisting of five anonymized clinical samples with known pathogenic cytogenetic abnormalities and three additional negative control samples without known pathogenic CNV, were analyzed in a blinded manner by the WES read depth model and aneuploidy analysis. ExomeDepth background read distribution was generated using only these eight samples.

A second group of fifty-three subjects from NCGENES who had clinical microarray testing at UNC prior to study enrollment were used for the CNV validation. CNVs detected using the Affymetrix CytoScan HD microarray platform (with a minimum size cut-off of 10 kb) were considered “known” CNVs for comparison to WES read depth-based methods. The specific microarray CNV calls and WES generated calls were compared to estimate the sensitivity and specificity of WES generated calls of different sizes.

In our third analysis, CNVs were predicted from all of the NCGENES samples using ExomeDepth. For each sample, the algorithm selected representative samples from all 672 samples in the study with similar read count distributions to optimize the background read count distribution. Variants identified were annotated with Bayes Factors, number of exons included in call region, allelic read fraction of SNPs within the CNV region, CNV size, and annotations from ISCA, DGV, RefSeq, and OMIM. Variants relevant to the corresponding patients’ phenotype were prioritized and analyzed concurrently with SNVs during molecular sign-out meetings for the NCGENES study. In some cases, CNVs were confirmed with an appropriate clinical test (e.g. MLPA) while in others we were able to opportunistically utilize an Illumina GSA array being run for a separate research study.

## Results

### Validation of CNV detection method

In the pilot study, all five pathogenic CNVs (Table 1) were accurately identified and three samples without pathogenic CNV were correctly identified as lacking pathogenic chromosomal anomalies. For four of the five pathogenic CNVs, breakpoints were very similar for the two detection methods. The limited overlap of one CNV call can be attributed to a lack of WES coverage in the non-overlapping regions.

**Table 1:** Pathogenic CNVs analyzed in a pilot study correctly identified by ExomeDepth and ploidy analysis.

Pathogenic CNV	Affymetrix CytoScan^a^ (size)	ExomeDepth^a^ (size)	Similarity to array^b^
1q21.1 microdeletion	chr1:146,105,170–147,844,758x1 (~1.7 Mb)	chr1:146,317,466–147,415,553x1 (~1.1 Mb)	63.1% ^c^
7q11.23 duplication	chr7:72,643,631–74,142,190x3 (~1.5 Mb)	chr7:72,717,454–74,133,478x3 (~1.4 Mb)	94.5%
15q11.2 microdeletion (Prader-Willi or Angelman Syndrome)	chr15:22,770,421–28,823,721x1 (~6.1 Mb)	chr15:22,833,303–28,525,580x1 (~5.7 Mb)	94.0%
17p12 duplication (Charcot-Marie Tooth type 1A)	chr17:14,098,660–15,501,133x3 (~1.4 Mb)	chr17:14,110,062–15,492,341x3 (~1.4 Mb)	98.5%
Trisomy 21	NA; 47,XY,+21 (by karyotype)	chr21x3 (by ploidy analysis)	NA

^a^ Coordinates based on hg19

^b^ Similarity computed using the Jaccard Similarity Coefficient (basepairs in intersection of CNV call and ISCA CMA variant / basepairs in the union of CNV call and ISCA CMA variant)

^c^ Lack of region coverage in the WES capture limited the accuracy of breakpoint identification.

NA = Not Applicable

Next, we used information from patients who had clinical CMA testing prior to enrollment in the NCGENES study to estimate the clinical sensitivity of ExomeDepth for CNV detection (Table 2). Of the 438 gold standard microarray CNV calls in the 53 patients with microarray data, 301 of these CNVs overlapped a region captured by WES and were thus considered “potentially detectable” by WES. The remaining 137 that did not overlap with a WES capture region were excluded from further analysis. When comparing all potentially detectable CNVs between the two methods (N = 301), ExomeDepth performed rather poorly, only reaching a sensitivity of ~40% for deletions and ~30% for duplications. Of the 208 CNVs that were not detected by ExomeDepth, 185 (90%) were in highly polymorphic regions. While ExomeDepth has been reported to be better than most methods at detecting CNV in polymorphic areas [4], our results confirm the known limitation of read depth-based CNV detection methods caused by high read depth variation in the selected background samples. However, by nature of their presence in a large proportion of the general population, most CNVs in these regions have little clinical relevance and therefore this technical limitation does not significantly impact clinical sensitivity. Limiting the microarray CNV calls to those that were not detected in multiple patients (N = 43), sensitivity for both deletions and duplications increases to 80% for deletions and 45% for duplications.

**Table 2:** Performance of WES-based CNV detection for “known” CNVs detected by clinical microarray.

	CMA Deletions ^a^	CMA Duplications ^a^	Deletion Sensitivity (%)	Duplication Sensitivity (%)
**All Microarray CNVs (N = 301)**	17/43	79/258	39.5	30.6
**Non-repeated CNVs (N = 43)** ^b^	8/10	15/33	80	45.5
**Non-repeated CNVs, adequately covered by WES (N = 32)** ^c^	8/9	15/23	88.9	65.2

^a^ Gold standard CNV calls from clinical microarray testing included deletions and duplications > 10 KB.

^b^ Limited to CNVs that were unique to a single patient within the cohort of 53

^c^ Limited to CNV regions overlapping coding genes that were not uncharacterized loci.

Because WES-based methods may not allow accurate discovery of CNV in intergenic regions, intronic regions, or in genes with poor WES capture, ExomeDepth may miss entire CNVs or inaccurately call CNV breakpoints in these regions. This phenomenon was observed in a female patient who had a VUS reported from previous CMA testing that was not observed in the WES data. CMA testing was able to detect a duplication involving three exons of the *SHOX* gene, but the WES methods did not make a call because the mostly intergenic variant only overlapped three exons that had poor coverage in many other samples. Importantly, this sample was originally analyzed using the SureSelect All Exon V4, and coverage improved in samples analyzed with the SureSelect All Exon V5 (See S1 Fig).

Since we would only consider a WES-based test responsible for the fraction of the genome targeted by the capture regions, we examined the performance of ExomeDepth after eliminating CNVs that only contained uncharacterized loci or were located in areas where WES coverage was severely limited in our test comparison. This further refined the sensitivity estimate of the WES method against the microarray “known” CNV set. All deletions except for one encompassing a pseudogene were correctly predicted, and 65% of duplications were correctly identified.

### NCGENES prospective CNV prediction

**Fig 1:**
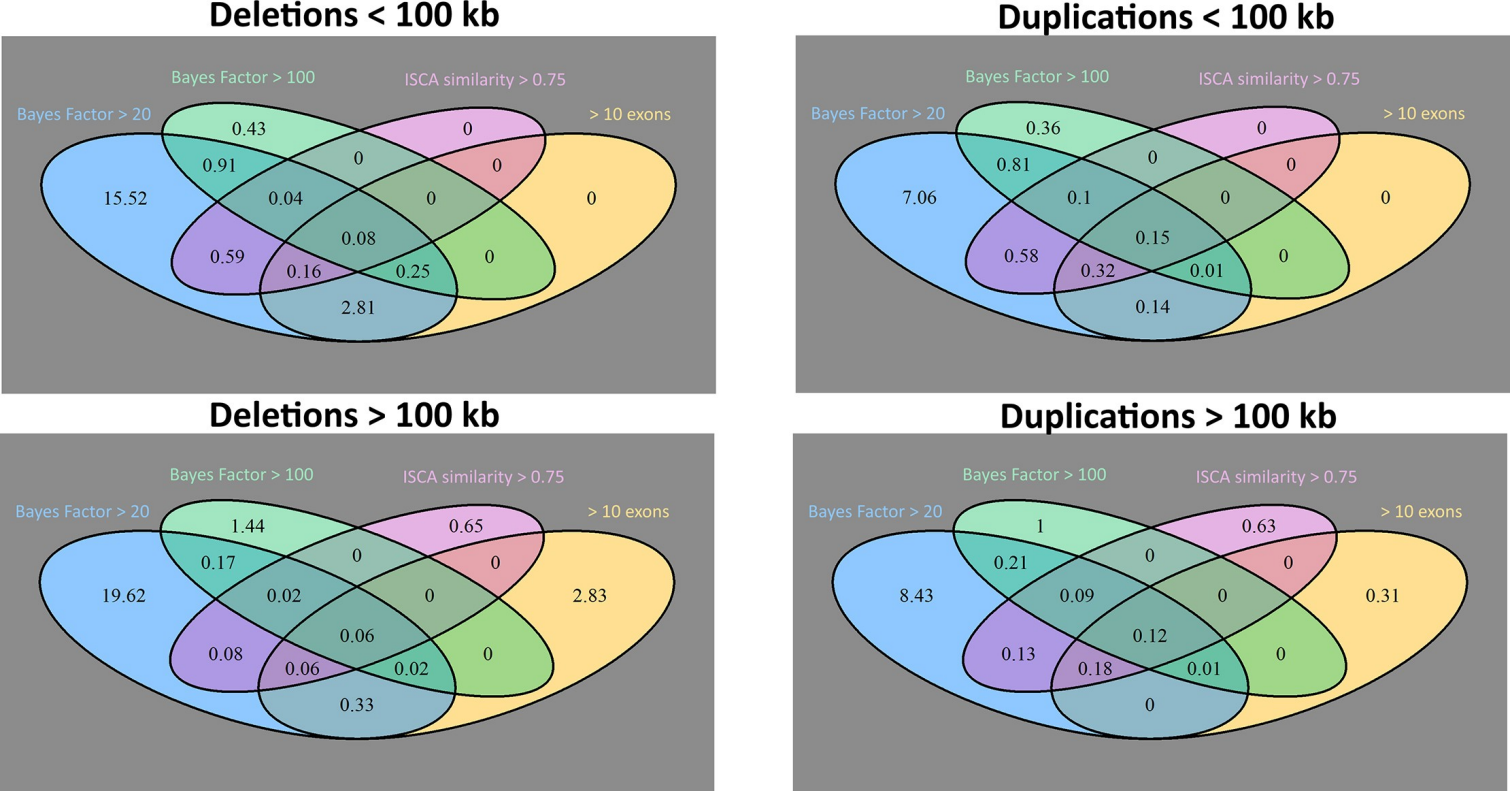
Mean number of deletions and duplications per person meeting filtering criteria. Top panels compare deletions and duplications < 100 kb. Bottom panels compare CNV > 100 kb. Left panels show numbers of deletions and right panels show duplications. Overall, there are more deletions with high Bayes factor scores per person than duplications with high Bayes factor scores. However, more duplications were detected that met multiple filtering criteria. Additionally, predicted CNV with a size > 100 kb were more likely to meet ISCA and number of exon criteria.

In addition to the retrospective analysis, CNV and aneuploidy detection methods were run prospectively on all 672 subjects from the NCGENES study. Raw output from ExomeDepth identified an average of 376 predicted CNVs per person (see S1 Table). However, very few of these variants had sufficient statistical and/or clinical significance to warrant further analysis. In smaller CNVs encompassing genes with less clinical or diagnostic relevance, using Bayes factors to assess the predicted variant’s level of statistical support eliminated the vast majority of potential CNVs. There were 29.6 CNV calls per person with Bayes factors >20 and 3.9 CNV calls per person with Bayes factors >100. Most of the remaining CNVs with higher Bayes factors could be adjudicated with other metrics including ISCA variant similarity, predicted CNV size, frequency of occurrence in other samples from the study, allelic read fractions of SNPs within the CNV region or number of exons involved (Fig 1). In particular, we found that the size of the CNV call was an especially useful metric to prioritize CNV calls from both a statistical and clinical standpoint due to its correlation with the number of coding regions within the predicted CNV.

Analysis of high priority CNVs (based on Bayes factor, ISCA classification, genes within region, and size) yielded the detection of eleven CNVs with potential diagnostic significance (described in Table 3) including seven deletions (size range: 2 exons to 3.8 Mb) and four duplications (size range: 416 kb to 1.2 Mb). Among these findings were microdeletions and microduplications having substantial phenotypic overlap to the patients’ clinical features as well as smaller deletions of only a few exons that provided a definitive molecular diagnosis. For example, a patient with a personal and family history of colorectal cancer with tumor studies indicative of Lynch syndrome (microsatellite instability and loss of *MLH1* by immunohistochemistry) was predicted by our analysis to have deletion of exons 2 and 3 of *MLH1*. Lynch syndrome was the leading clinical diagnosis, and the two exon deletion was confirmed clinically with MLPA (Multiplex Ligation-dependent Probe Amplification). Additionally, a patient with a clinical diagnosis of either cone or cone-rod dystrophy was found to have an approximately 85 kb deletion of the *CRX* gene, which is implicated in autosomal dominant cone-rod retinal dystrophy 2, a very good fit for the patient’s phenotype. This deletion was subsequently confirmed clinically by qPCR.

**Table 3:** CNVs of potential diagnostic significance detected in NCGENES patients.

Clinical details and previous work-up	CNV finding (size)	Predicted Breakpoints^a^	Interpretation^b^	Notes; Follow Up
29 year-old female with retinitis pigmentosa (RP); WES identified apparently homozygous splice site variant in *MERTK*	2q13 x1 (~1.7 Mb)	chr2:111395390–113090216	KP (for RP) VUS (for 2q13 deletion syndrome)	Reported as the presumed etiology due to compound heterozygous large deletion and splice site variant; confirmed by Illumina GSA array
32 year-old male with colorectal cancer, suspected Lynch syndrome (tumor studies positive for microsatellite instability and loss of MLH1 by IHC)	MLH1 ex2-3 x1 (~4.6 kb)	chr3:37038036–37042654	LP	Reported as likely etiology; clinically confirmed by MLPA
10 year-old male with hypotonia, intellectual disability, autistic features; normal karyotype and fragile X	3q13.2–13.31 x1 (~3.8 Mb)	chr3:110994278–114833200	LP	3q13.31 microdeletion syndrome (OMIM #615433) is considered a likely etiology for the patient’s symptoms; confirmed by Illumina GSA array
14 year-old male with microcephaly, generalized epilepsy, intellectual disability, tremor, hearing loss, mild dysmorphic features; normal BAC microarray in 2006	6q22.1-q22.31 x1 (~3.8 Mb)	chr6:116575201–120336357	LP	6q22 microdeletion syndrome reported as a likely etiology; clinically confirmed by microarray
Encephalopathy, intellectual disability, hypotonia, generalized joint hypermobility, ataxia, and fatigue; Negative genetic testing for myotonic dystrophy, Fragile X, Prader-Willi and negative mitochondrial DNA SNV and del/dup testing	8q24.3 x3 (~0.4 Mb)	chr8:145532476–145948790	VUS	Previously known by clinical array; Larger CNVs have been associated with facial dysmorphism and intellectual disability [18,19], this smaller duplication might narrow the critical region
53-year-old female with adult-onset muscle weakness and elevated creatine kinase, mild sensory neuropathy; muscle biopsy nonspecific	13q12.12 x3 (~1.1 Mb)	chr13:23777767–24895797	VUS	Copy number gain encompassing *SACS* and *SGCG*, both of which are implicated in neuromuscular disorders, considered a possible etiology; confirmed by Illumina GSA array
13-year-old female with intellectual disability, epilepsy, autism spectrum disorder; Normal BAC array in 2006	16p11.2 x1 (~0.5 Mb)	chr16:29674985–30199927	KP	16p11.2 deletion syndrome (OMIM #611913) reported as a likely etiology; clinically confirmed by microarray
Dystonia and extreme spasticity. Previously thought to have Russell-Silver Syndrome	17q12 x3 (~0.4 Mb)	chr17:32482871–32908195	VUS	Overlapping with 17q12 microduplication syndrome—highly variable presentations usually including intellectual disability and developmental delay [20]
3-year-old male with cholestasis, hypercholestesterolemia, xanthomas, chronic liver failure, and developmental delay	19p13.3 x1 (~0.5 Mb)	chr19:959958–1440348	LP	19p13.3 contiguous gene syndrome including *STK11* (Puetz-Jeghers syndrome); provides a partial explanation but no reports of cholestasis/liver failure with this deletion; confirmed by Illumina GSA array
40-year-old male with cone dystrophy; nondiagnostic results through clinical sequencing	*CRX* x1 (~85 kb)	chr19:48304924–48389590	KP	Cone-rod dystrophy (OMIM #120970) reported as the presumed etiology; clinically confirmed by qPCR
6-year-old female with intellectual disability, epilepsy, behavioral difficulties; Metabolic work-up identified 3-methylglutaconic aciduria	22q11.23 x3 (LCR F-H) (~1.3 Mb)	chr22:23735752–24989043	VUS	22q11.23 distal duplication syndrome (LCR F-H) reported as a possible etiology; clinically confirmed by microarray

^a^ Coordinates based on hg19

^b^ KP,LP, and VUS represent for known pathogenic, likely pathogenic, and variant of unknown significance

We detected chromosomal aneuploidy in three patients. One individual was enrolled due to an aortic aneurysm at age 32, and had a previously known karyotype consistent with Klinefelter syndrome (47,XXY). Another patient with Down syndrome due to trisomy 21 was enrolled in the study to evaluate intractable seizures and a neurodegenerative disorder with loss of milestones (which are clearly atypical for Down syndrome). In addition to gain of the entire chromosome 21, exome sequencing identified a missense variant in the *GABRG2* gene (c.919T>G [p.L307V]) that was found to be *de novo* upon testing of parental samples, and provides a plausible explanation for the unusually severe neurological phenotype in this patient. Finally, we detected a case of mosaicism (coverage of 50% of expected values across all covered regions of the Y chromosome) likely indicating somatic loss of the Y chromosome (LOY). This finding was made in a 61-year-old man with pheochromocytoma and renal cancer, who is also a lifelong smoker. Interestingly, there have been recent connections between smoking status, somatic LOY in peripheral blood samples, and non-hematologic cancers [21,22], raising the possibility that this finding could be related to the patient’s cancer diagnoses.

This analysis also identified a medically actionable secondary finding. A 38-year-old male who was initially enrolled in the NCGENES study for cardiomyopathy was found to have a 27 kb whole gene deletion of *MSH6* (hg 19, chr2:48010242–48037615). According to the ACMG recommendation for secondary findings [23] and our own definition of medical actionability [24], this result is considered a reportable incidental finding and was therefore confirmed clinically by MLPA before being returned to the study participant.

Additionally, we identified ten CNVs of possible relevance to the patient’s phenotype but enough uncertainty regarding that relevance that they were designated VUS (Table 4). In six of these individuals, CNVs previously reported as pathogenic were identified in individuals with little or no phenotypic overlap with the previously reported syndromes. These likely represent examples of incomplete penetrance for these CNVs. In another eight individuals, we identified microdeletions or microduplications involving the 15q11.2 breakpoint 1 and 2 regions (BP1 and BP2) (S2 Table), most of whom did not have phenotypic features consistent with reports in the literature. In the prospective NCGENES analysis, all 12 chromosomal abnormalities with clinical follow up (all CNV from Table 1 and 2q31.2 duplication in Table 4) have been confirmed, ranging from a two exon deletion to whole chromosome duplications. Overall, these results correspond to an additional diagnostic rate of 1.6% and the identification of a CNV of interest in around 3.3% of sequenced samples. These cases illustrate that while most detectable pathogenic CNV from this WES detection method are large, smaller clinically relevant CNV can be detected as well.

**Table 4:** CNVs with unknown clinical significance due to uncertain pathogenicity or unclear phenotypic overlap.

Clinical details and previous work-up	CNV finding (size)	Predicted Breakpoints^a^	Interpretation^b^	Notes; Follow Up
Clinical diagnosis of von Willebrand disease	1q21.1 x1 (~1.1 Mb)	chr1: 146317466–147415637	KP	Interpreted as being probably non-penetrant for 1q21.1 deletion syndrome
23-year-old male with mild intellectual disability, spasticity, and motor and sensory neuropathy clinically diagnosed as Charcot Marie Tooth type V	2q31.2 x3 (~0.5 Mb)	chr2:179300822–179839937	VUS	Novel contiguous gene duplication encompassing TTN. Confirmed by Illumina GSA array.
Intellectual disability; focal nodular hyperplasia of the liver	10q23.1–23.2 x3 (~6.9 Mb)	chr10:82095766–88975883	VUS	Known from previous testing. Relatively gene poor region.
Weakness, fatigue, and autonomic dysfunction–Suggested, but uncertain mitochondrial myopathy by muscle biopsy; family history of fatigue, seizure-like episodes, and autonomic dysfunction	16p11.2 x3 (~1.8 Mb)	chr16:28426026–30199845	VUS	Possibly contributory. 16p11.2 microduplication syndrome would not completely explain patient’s phenotype.
Postpartum cardiomyopathy; heart failure	16p11.2 x3 (~0.5 Mb)	chr16:29674985–30199845	VUS	Interpreted as likely non-penetrant as patient has no findings suggestive of 16p11.2 microduplication syndrome.
Breast Cancer	16p13.11 x1 (~0.8 Mb)	chr16:15489748–16292070	KP	Patient’s phenotype not compatible with typical findings of 16p13 deletion syndrome (intellectual disability, ADHD, epilepsy, schizophrenia), interpreted as non-penetrant
Non-ischemic dilated cardiomyopathy	*MYH2 x1* (~25 kb)	chr17:10426308–10451418	LP (for AR proximal myopathy and ophthalmoplegia); VUS (for cardiomyopathy)	
Cone dystrophy, hearing loss, ADHD, dominant family history	17q12 x1 (~1.4 Mb)	chr17:34842566–36214231	LP	17q12 microdeletion syndrome [25]- developmental delay and autism, interpreted as non-penetrant
Progressive cerebellar ataxia and dystonia; Negative genetic testing for SCA 1,2,3,6,7,8,13,14,17 and Friedrich’s Ataxia	19q13.42 x3 (~0.6 Mb)	chr19:54135274–54723724	VUS	Previously known by clinical array, contains 19 genes and a cluster of miRNAs
Stargardt’s disease	22q11.21 x3 (LCR A-D) (~2.5 Mb)	chr22:18910276–21411676	LP	22q11.2 microduplication syndrome, Interpreted as non-penetrant

^a^ Coordinates based on hg19

^b^ KP,LP, and VUS represent for known pathogenic, likely pathogenic, and variant of unknown significance

In two patients, the use of ExomeDepth in conjunction with WES analysis identified two variants in a gene associated with an autosomal recessive disease. In a 29 year-old woman with retinitis pigmentosa, WES identified an apparently homozygous splice site variant in *MERTK* associated with retinitis pigmentosa (MIM **#**613862). Subsequent CNV analysis with ExomeDepth detected a ~1.7 Mb deletion involving this gene that was confirmed by Illumina GSA array (Table 3). This large heterozygous deletion is thus *in trans* with the splice site variant and these compound heterozygous variants are a better fit for the clinical scenario than a homozygous splice site variant, given a lack of evidence of consanguinity in the family.

**Fig 2:**
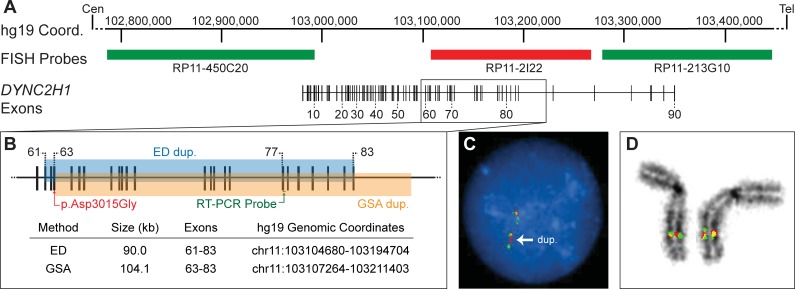
Orthogonal CNV detection methods confirm a DYNC2H1 duplication in an anomalous fetus. A. An intron-exon map of the DYNC2H1 gene is depicted with respect to hg19 genomic coordinates (note nearby genes are not indicated for simplicity). Also shown are the locations of the fluorescence in situ hybridization (FISH) probes (RP11-450C20, RP11-2I22, and RP11-213G10), the pathogenic SNV (red) identified by WES, the Real-Time PCR probe (green), and the approximate coordinates of the paternal duplication identified by ExomeDepth (ED) in aqua and by the GSA array in orange. B. An interphase FISH image shows an enhanced/duplicated red signal flanked by a green signal on either side, indicative of a tandem duplication. C., D. Metaphase FISH analysis shows an enhanced red signal, representing the duplicated region, isolated to chromosome 11. Panel D shows an isolated view of both chromosome 11 homologs from a second metaphase cell. DAPI stain was converted to black and white for better visualization of red and green signals.

The second case was evaluated as part of a trio study of fetal anomalies [26] from an ongoing prenatal whole exome study at UNC-CH in which the fetus presented clinically during the second trimester with fetal skeletal malformations suggestive of short-rib polydactyly. On analysis of WES variant data, a single heterozygous maternally inherited known pathogenic SNV was identified in the *DYNC2H1* gene, c.9904A>G (p. Asp3015Gly) [27,28]. Although this gene is associated with autosomal recessive inheritance of short-rib thoracic dysplasia (MIM #613091) and only one heterozygous variant was found, the high degree of phenotypic overlap suggested that the second allele may have been missed. We therefore used ExomeDepth analysis in the trio and identified a ~90 kb duplication within the *DYNC2H1* gene in the fetal and paternal samples. Presence of the duplication was confirmed by qPCR and Illumina GSA Array. Fluorescence in situ hybridization (FISH) analysis was consistent with this interpretation (Fig 2A). FISH analysis also confirmed that the duplication occurs near the innate location of the *DYNC2H1* gene on chromosome 11 and likely represents a tandem duplication (Fig 2B–2D). While precise breakpoints have not been identified, the ~90kb duplication appears to represent a disruptive intragenic duplication present *in trans* with the known pathogenic SNV.

## Discussion

### WES-based CNV diagnostic testing

These results demonstrate that CNV detection from WES read depth data in a cohort unselected for cytogenetic abnormalities can effectively identify clinically relevant CNVs and expand the diagnostic yield of WES. While there are definite limitations that restrict its use as a gold-standard diagnostic test for CNVs, our data show that opportunistic analysis of WES data may increase the diagnostic yield by 1–2% when used as a second-line test after CMA. When combined with traditional WES as first-line test, we expect that the yield would be even higher. Given that ExomeDepth performs very well for the detection of large CNVs responsible for recurrent deletion/duplication syndromes and aneuploidy, one might expect that WES analysis including CNV detection would outperform CMA in a prospective head-to-head comparison. Indeed, our diagnostic rate of 1.6% with CNV testing appears to be consistent with the diagnostic rate observed with clinical CMA methods in similar testing circumstances. While as a first-tier test CMA analysis has positive rates around 15–20% [29], the diagnostic rate for CMA in cohorts who have already had some genetic testing appears to be somewhere between 2.4 and 10% [7, 30]. The lower level observed in this study could be attributed to the fact that, prior to enrollment in the NCGENES study, most participants from the high yield cohorts for CNV testing such as developmental delay already a normal karyotype and negative CMA testing. Furthermore, the NCGENES cohort included several different phenotypic sub-groups (e.g. Hereditary Cancer) among which we would not expect to find an excess of pathogenic CNVs. Lastly, our use of ExomeDepth and most of our annotation was chosen in an effort to minimize false positive results which, while not noted to be problematic on a clinical level in our pilot studies, could theoretically increase the false negative rate as well. Interestingly, in several cases coverage analysis of WES data in NCGENES participants identified clinically relevant CNVs that were missed by previous clinical microarray testing using older BAC array technology, in which limited backbone coverage was available, or regions responsible for certain recently described genomic disorders were not included.

### Known pathogenic CNVs in patients with discordant phenotypes

Several pathogenic CNVs were detected in individuals whose phenotypes were inconsistent with the conditions caused by those CNVs, suggesting incomplete penetrance or possibly a broader phenotype than is currently associated with these known pathogenic CNVs. In our NCGENES data set, the majority of participants did not have CNV testing as part of their clinical workup because their phenotypes did not warrant this type of testing. However, in this cohort, we have found fourteen CNVs previously reported as pathogenic including eight 15q11.2 BP1-BP2 deletions or duplications (see S2 Table), DiGeorge syndrome region duplications, CNVs related to autism spectrum disorder, and others. The presence of these CNVs in unaffected adults provides additional evidence of the incomplete penetrance and variable expressivity described for many of these variants. Discovery of these variants raises the question of whether these findings should be reported in a diagnostic setting, when they provide no additional diagnostic insight but might be relevant to the patient’s risk to develop symptoms in the future (e.g. 22q11.2 CNV and risk for schizophrenia) or reproductive risks given that they may pose a risk for disease in offspring or other members of the family in the case of a familial CNV. One concern in this situation is that prediction of phenotypic consequences in offspring is challenging, given the reduced penetrance, variable expressivity and frequent lack of identifiable features clinically.

### CNV detection shortfalls and filtering

We find that ExomeDepth, accurately identifies large, clinically relevant CNVs. However, because of its reliance on comparisons to other samples, it may not accurately predict copy number in highly polymorphic regions where there are divergent copy numbers among control samples. This is reflected in the improvement of ExomeDepth sensitivity when comparisons are restricted to non-polymorphic regions of the genome. Additionally, the many CNVs that do not include exonic regions are not detectable with a WES-based test. While some known CNVs were not detected for this reason, this limitation is shared by WES testing in general and is mitigated by the fact that most clinically relevant CNV includes a portion of the coding region of the genome.

Also, read count methods inherently favor the accurate discovery of deletions as opposed to duplications [5,7,11]. Our data support this finding, with substantially higher sensitivity for deletions compared to duplications. In addition, the ISCA database for structural variation [15] contains roughly the same number of deletions and duplications overall, but has about 1.75 times more pathogenic deletions than pathogenic duplications. This finding reflects the difficulty in assessing whether gains in copy number (triplosensitivity) for certain regions are as deleterious as copy number loss (haploinsufficiency). Therefore, missing a duplication call may be less problematic than missing a deletion. Still, the detection rate of variants should improve with increased mean depth of coverage, which was a limitation of our research-based exome sequencing.

Lastly, although the specificity of raw ExomeDepth data would currently make it inadequate for routine clinical use without a secondary confirmation method, filtering CNV calls based on size and number of exons greatly improves specificity and can lead to the accurate discovery of pathogenic or other CNVs of interest. Previously published data has shown that this CNV prediction method and other similar methods have very high false discovery rates above 85% [6] and possibly as high as 97% [7] for single exon calls. As suggested by our pilot study, deletions do have a higher confirmation rate compared to duplications with a false discovery rate as low as 22%. We did not systematically evaluate all calls made by ExomeDepth, but considering only calls encompassing multiple exons with additional supporting statistical evidence, such as a high Bayes factor, all pathogenic CNV that have been clinically tested were validated.

## Conclusion

The opportunistic analysis of CNVs predicted by WES read depth data serves as a highly useful adjunct screen for clinically relevant CNVs in the exome and has the potential to increase diagnostic yields. WES-based methods should not be used for primary diagnostic CNV analysis at this time, and smaller or less confidently called CNVs should be interpreted with proper skepticism and confirmed with orthogonal methods. However, in patients undergoing WES testing, additional analysis of CNVs may allow for the accurate discovery of most large, pathogenic CNVs and many smaller CNVs related to the patient’s phenotype.

## Supporting information

S1 Fig SHOX read depth for patient with CMA detected duplication. Box blot of read coverage over SHOX exons corrected for total number of reads per sample. Red diamond shows read depth of patient with CMA detected duplication. Low coverage of SHOX by some samples including the patient with a CMA detected duplication could be explained by poor capture of this region by SureSelect All Exon V4.
(TIF)

S1 Table Mean number of ExomeDepth CNV predictions per person from 672 exomes. Number of predicted deletions, duplications, and total variants meeting different filtering criteria based on predicted Bayes Factor, similarity to known pathogenic variant in ISCA database, and variant size.
^a^ Bayes Factor here is a likelihood ratio of CNV to normal copy number state. E.g. Bayes Factor of 20 for a heterozygous deletion indicates that it is 20 times more likely given the WES data for that region that this stretch of the genome has one copy as opposed to two.
^b^ Similarity computed using the Jaccard Similarity Coefficient (basepairs in intersection of CNV call and ISCA variant / basepairs in the union of CNV call and ISCA variant)
(DOCX)

S2 Table 15q11.2 BP1–BP2 gains and losses predicted in NCGENES patients. 15q11.2 deletions predicted in NCGENES patients largely inconsistent with known phenotype. The 15q11.2 duplication syndrome has been associated with developmental delay, dysmorphic features, autism, and seizures. The deletion syndrome has been associated with susceptibility to neuropsychiatric or neurodevelopmental problems and seizures.
^a^ Coordinates based on hg19
(DOCX)

S1 Appendix Supplemental_python_scripts.tar.gz. Compressed archive of in-house python scripts used to annotate exomeDepth raw CNV predictions. A README file is included in archive.
(GZ) Click here for additional data file.

S1 Dataset NCGENES_exD_results_deidentified.csv.gz. Compressed comma-separated file containing annotated CNV predictions from de-identified subjects from the NCGENES study.
(GZ) Click here for additional data file.
