# Supplementary figures and images for: Increasing the diagnostic yield of exome sequencing by copy number variant analysis
Source: PLoS One. 2018 Dec 17;13(12):e0209185. doi: 10.1371/journal.pone.0209185 (PMC6296659; doi:10.1371/journal.pone.0209185)

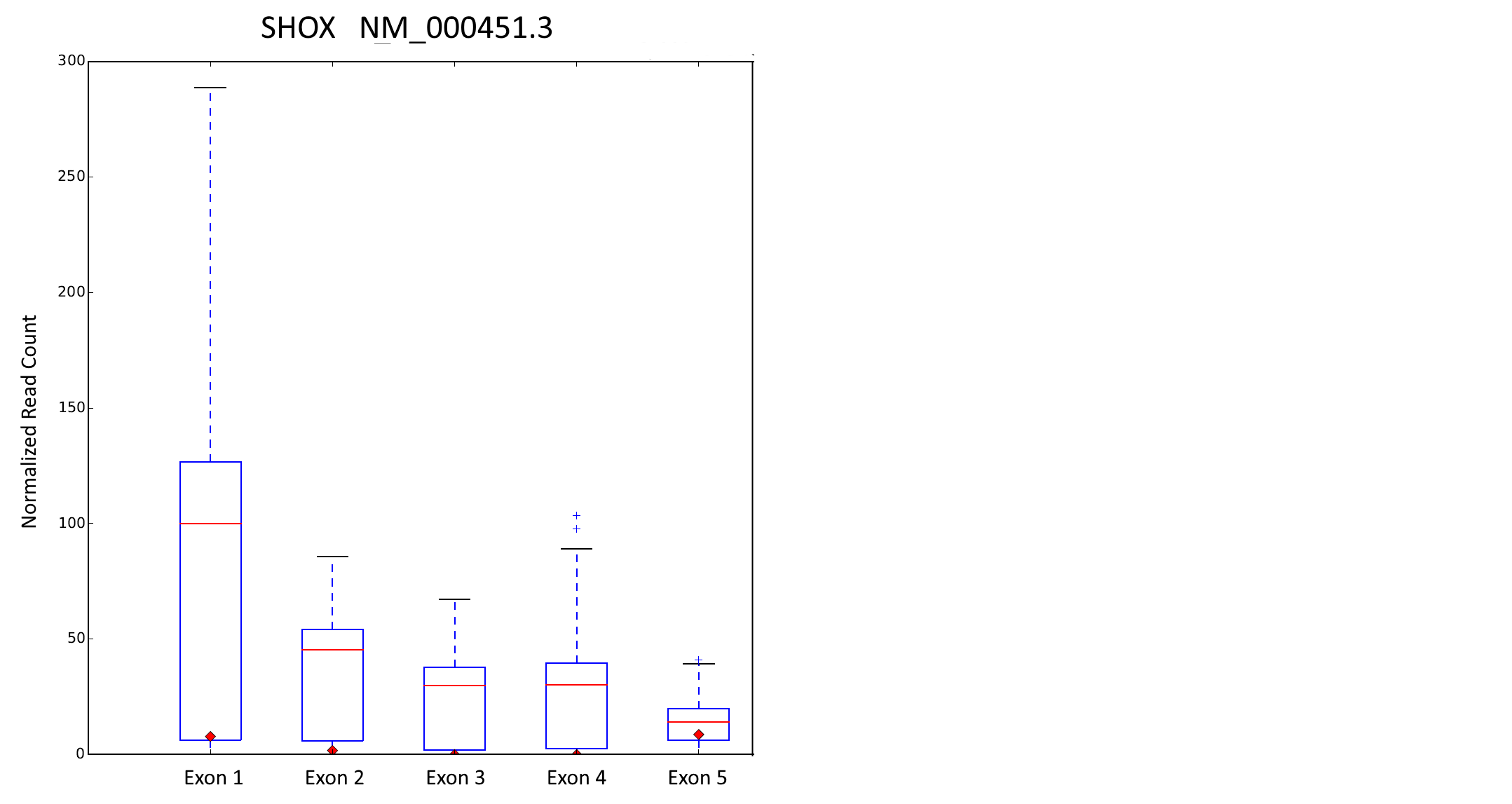

Supplement: S1 Fig — Box blot of read coverage over SHOX exons corrected for total number of reads per sample. Red diamond shows read depth of patient with CMA detected duplication. Low coverage of SHOX by some samples including the patient with a CMA detected duplication could be explained by poor capture of this region by SureSelect All Exon V4. (TIF) [file pone.0209185.s001.tif]
