# Supplementary material for: Increasing the diagnostic yield of exome sequencing by copy number variant analysis
Source: PLoS One. 2018 Dec 17;13(12):e0209185. doi: 10.1371/journal.pone.0209185 (PMC6296659; doi:10.1371/journal.pone.0209185)
Supplement: S1 Table — Number of predicted deletions, duplications, and total variants meeting different filtering criteria based on predicted Bayes Factor, similarity to known pathogenic variant in ISCA database, and variant size. a Bayes Factor here is a likelihood ratio of CNV to normal copy number state. E.g. Bayes Factor of 20 for a heterozygous deletion indicates that it is 20 times more likely given the WES data for that region that this stretch of the genome has one copy as opposed to two. b Similarity computed using the Jaccard Similarity Coefficient (basepairs in intersection of CNV call and ISCA variant / basepairs in the union of CNV call and ISCA variant) (DOCX) [file pone.0209185.s002.docx]

**Supplemental Table 1.** Mean Number of ExomeDepth CNV calls per person from 672 exomes.

| **CNV type** | **Number predicted CNV** | **Bayes Factor^a^ > 20** | **Bayes Factor^a^ > 100** | **Similarity^b^ to ISCA variant > 75%** | **Size > 100kb** | **Number exons > 10** | **Meeting all criteria** |
| --- | --- | --- | --- | --- | --- | --- | --- |
| Deletions | 269.5 | 20.4 | 3.3 | 1.7 | 6.4 | 0.9 | 0.1 |
| Duplications | 106.6 | 9.2 | 0.6 | 1.4 | 4.0 | 1.1 | 0.1 |
| Total | 376.1 | 29.6 | 3.9 | 3.1 | 10.4 | 2.0 | 0.2 |

^a^ Bayes Factor here is a likelihood ratio of CNV to normal copy number state. E.g. Bayes Factor of 20 for a heterozygous deletion indicates that it is 20 times more likely given the WES data for that region that this stretch of the genome has one copy as opposed to two.

^b^ Similarity computed using the Jaccard Similarity Coefficient (basepairs in intersection of CNV call and ISCA variant / basepairs in the union of CNV call and ISCA variant)
