# Supplementary material for: Increasing the diagnostic yield of exome sequencing by copy number variant analysis
Source: PLoS One. 2018 Dec 17;13(12):e0209185. doi: 10.1371/journal.pone.0209185 (PMC6296659; doi:10.1371/journal.pone.0209185)
Supplement: S2 Table — 15q11.2 deletions predicted in NCGENES patients largely inconsistent with known phenotype. The 15q11.2 duplication syndrome has been associated with developmental delay, dysmorphic features, autism, and seizures. The deletion syndrome has been associated with susceptibility to neuropsychiatric or neurodevelopmental problems and seizures. a Coordinates based on hg19 (DOCX) [file pone.0209185.s003.docx]

**Supplemental Table 2.** 15q11.2 BP1–BP2 gains and losses found in NCGENES patients. The duplication syndrome has been associated with developmental delay, dysmorphic features, autism, and seizures. The deletion syndrome has been associated with susceptibility to neuropsychiatric or neurodevelopmental problems and seizures.

| Chromosomal location and CN status | Predicted  Breakpoints^a^ | Patient’s Clinical Details |
| --- | --- | --- |
| 15q11.2 x3 | chr15:22836043-23300241 | Seizures and developmental delay |
| 15q11.2 x3 | chr15:22835843-23052673 | Neuropathic pain and weakness in lower extremity |
| 15q11.2 x1 | chr15:22833403-23300241 | Seizures and developmental delay |
| 15q11.2 x3 | chr15:22833303-23300312 | Skeletal dysplasia and developmental delay |
| 15q11.2 x1 | chr15:22833403-23300312 | Colon and ovarian cancer |
| 15q11.2 x3 | chr15:22833303-23182054 | Autism, developmental delay, and dysmorphic features |
| 15q11.2 x1 | chr15:22833303-23182054 | Platelet function disorder |
| 15q11.2 x3 | chr15:22440530-23300141 | Neutropenia, thrombocytopenia, and microcephaly |

^a^ Coordinates based on hg19
